# Citri reticulate pericranium-derived extracellular vesicles exert antioxidant and anti-inflammatory properties and enhance the bioactivity of nobiletin by forming EVs-nob nanoparticles

**DOI:** 10.3389/fcell.2024.1509123

**Published:** 2024-12-23

**Authors:** Ling Ma, Zimao Ye, Dongqin Guo, Chao Nie, Zhiqin Zhou

**Affiliations:** ^1^ College of Pharmacy, Chongqing Three Gorges Medical College, Chongqing, China; ^2^ Key Laboratory of Agricultural Biosafety and Green Production of Upper Yangtze River (Ministry of Education), College of Horticulture and Landscape Architecture, Southwest University, Chongqing, China; ^3^ The Southwest Institute of Fruits Nutrition, Chongqing, China

**Keywords:** citri reticulate pericranium, extracellular vesicles, antioxidant, anti-inflammatory, nobiletin

## Abstract

Plant-driven extracellular vesicles (PEVs) have attracted significant interest due to their natural origin, remarkable bioactivity, and efficacy in drug encapsulation and target delivery. In our work, extracellular vesicles from Citri Reticulate Pericranium (CEVs) were isolated and investigated their physicochemical characteristics and biological activities. We identified the vesicle structures as regular, with a particle size of approximately 200 nm. We also detected large quantities of lipids, proteins, carbohydrates, as well as total phenols and total flavonoids. The rich content of CEVs endows them with significant antioxidant and anti-inflammatory effects, which can notably increase the levels of GSH and effectively promote the activity of antioxidant markers such as SOD, CAT, and GR. Additionally, they can inhibit the level of inflammatory markers like NO and inflammatory biological factors (*IL-6*, *IL-1β* and *TNF-α*). In addition, we successfully synthesized EVs-nob nanoparticles with a 83.75% ± 2.83% encapsulation rate and 2.79% ± 0.02% drug loading, which may enhance the antioxidant and anti-inflammatory effects of nobiletin. Our research provides critical insights into the bioactivity of CEVs and demonstrates the significant potential of PEVs in nanocarrier creation, thereby promoting the advancement of more PEVs for biomedical applications.

## 1 Introduction

Extracellular vesicles (EVs) are a diverse set of nanoscale vesicles (varying in diame-ter from 30 to 1,000 nm) produced by cells and released by almost all eukaryotic and prokaryotic cells (Kalra et al., 2016; Lian et al., 2022; Raposo and Stoorvogel, 2013). They comprise a lipid and glycoprotein bilayer structure with metabolites, proteins, lipids, nucleic acids, and other cargo therein (Li et al., 2021; Colombo et al., 2014; Zhang et al., 2015). They can transport cargo to destination cells and act as extracellular messengers, facilitating inter-cellular communication (Wortzel et al., 2019; Lopez-Verrilli and Court, 2013; Ludwig and Giebel, 2012).

In recent decades, exosomes generated from mammalian cells, a category of extracellular vesicles, have been extensively utilized in medicine, including diagnostics, disease modification, and medication administration (He et al., 2018; György et al., 2015; Kalluri and LeBleu, 2020). Recent studies have increasingly demonstrated the presence of plant-derived extracellular vesicles that exhibit characteristics akin to mammalian exosomes (Halperin and Jensen, 1967; López De Las Hazas et al., 2023; Shao et al., 2023). Plant cell-generated extracellular vesicles provide various benefits over exosomes formed from animal cells: 1) Natural botanical origin; 2) minimal toxicity and diminished immunological reaction; 3) economic manufacturing expenses. The intrinsic benefits have generated heightened interest in the development of plant-derived extracellular vesicles as a novel approach to health management (Fan et al., 2022; Rome, 2019). Thus, an increasing amount of research indicates that plant-derived nanovesicles can modulate many cellular abnormalities, demonstrating significant anti-inflammatory, antioxidant, and anti-tumor properties (Hansen and Nielsen, 2017; Liu et al., 2022; Raimondo et al., 2022; You et al., 2021; Zhang et al., 2023). Extracellular vesicles originating from ginger transport curcumin, which can specifically target inflamed colons and mitigate inflammation (Dad et al., 2021). Similarly, grape-derived extracellular vesicles can selectively target mouse intestinal stem cells, speeding intestinal epithelial cell regeneration and encouraging intestinal tissue remodeling, which can help prevent and alleviate colitis brought on by dextran sulfate sodium (Ju et al., 2013). Raimondo et al. discovered that extracellular vesicles obtained from lemons can selectively target tumor locations and impede the proliferation of multiple myeloma by facilitating the killing of tumor cells via the activation of the TRAIL signaling pathway (Raimondo et al., 2022). Furthermore, PEVs’ lipid bilayer structure allows for the development of novel types of nanoparticle carriers for drug encapsulation and delivery (Woith et al., 2021). Tomohiro Umezu et al. encapsulated miRNA within vesicles derived from the Barbados cherry (Malpighia emarginata DC.), successfully safeguarding miRNA and directing it to recipient cells (Umezu et al., 2021). Additionally, Xu et al. revealed that exosomes derived from ginseng can function as innovative nanoplat-forms for the delivery of microRNA and the promotion of neural development in stem cells (Xu et al., 2021). Ramila Mammadova and others employed ultrasound to encapsulate curcumin into tomato extracellular vesicles, therefore increasing its anti-inflammatory efficacy (Mammadova et al., 2023).

Citri Reticulate Pericranium (CRP), often known as “Chenpi,” is the dried mature fruit peel of Citrus reticulata Blanco and its cultivated varieties in the Rutaceae family, which includes citrus plants (Yu et al., 2018). CRP has been used as a medicinal plant in East and Southeast Asia for thousands of years (Zhang et al., 2024). In addition to its medical properties, it has been utilized as a food or a component in food production (Casquete et al., 2015; Remigante et al., 2023). More and more research has recently demonstrated that CRP has functional activities such as antioxidant, anti-inflammatory, antiviral, and anti-obesity properties (Barreca et al., 2011; Guo et al., 2016). A variety of bioactive compounds found in CRP, such as flavonoids, essential oils, polysaccharides, phenolic acids, and alkaloids, are responsible for these high bioactivities (Chen et al., 2022). Extracellular vesicles (EVs), a recently discovered class of bioactives, have received insufficient attention in terms of characterization and bioactivities. Furthermore, the bioactive substances in CRP change with storage time, and it is unclear how the activity of EVs changes with CRP storage duration.

The aim of this study was to extract EVs from CRP with varying storage times, analyze their composition and properties, investigate their potential role in alleviating oxidative stress and inflammation in biological cells, and assess their efficacy as drug carriers. CRP-derived EVs were extracted using PEG precipitation, and the study was divided into three parts: 1) characterization, compositional analysis, and chemical antioxidant activity testing of EVs; 2) evaluation of the ability of EVs to mitigate oxidative stress induced by H_2_O_2_ and reduce inflammation in LPS-induced RAW264.7 macrophages; and 3) encapsulation of nobiletin (nob) pigment into EVs as a carrier and assessment of the antioxidant and anti-inflammatory effects of EVs-nob on H_2_O_2_ and LPS-treated cells.

## 2 Reagents and methods

### 2.1 Materials and reagents

Citri Reticulate Pericranium was purchased in Chongqing Wanli Pharmaceutical Co., Ltd. Nobiletin was procured from Shaanxi Huike Botanical Development Co., Ltd. RAW264.7 mouse macrophages were procured from Wuhan Procell Life Science and Technology Co., Ltd. Lipopolysaccharides (LPS) were purchased from Nanjing Herbal Bio-technology Co., Ltd. Hydrogen peroxide (H_2_O_2_) was purchased from Chongqing Chuandong Chemical Co., Ltd. Dulbecco’s Modified Eagle Medium, DMEM purchased from Thermo Fisher Scientific. fetal bovine serum, FBS and Penicillin/Streptomycin purchased from Yeasen Biotechnology (Shanghai) Co. Ltd. Nitric oxide test kits were purchased in Beyotime (China). Malondialdehyde kit, glutathione kit, superoxide dismutase kit, catalase kit and glutathione reductase kit, purchased from Beijing Solarbio Science and Tech-nology Co., Ltd. RNA extract kit purchased from TIANGEN Biochemical Technology (Beijing) Co., Ltd. polyvinyl alcohol, PVA purchased from Chengdu Kelong Chemical Co., Ltd. PEG 6000 was purchased Chengdu Kelong Chemical Co., Ltd.

### 2.2 Extraction and characterization of EVs from CRP

#### 2.2.1 Extraction of EVs from CRP

The samples were soaked in ultrapure water for 24 h and then pulverized using a grinder. The resulting mixture was centrifuged at 4°C and 3,000 rpm for 10 min to remove tissue debris, collecting the supernatant. This supernatant was further centrifuged at 4°C and 5,000 rpm for 20 min. Subsequently, it was centrifuged again at 4°C and 13,000 rpm for 1 h and filtered through a 0.45 μm membrane to obtain a clear supernatant. This supernatant was mixed with PEG6000 solution to achieve a final concentration of 10% PEG6000, stirred thoroughly, and incubated at 4°C overnight. After incubation, the mixture was centrifuged at 13,000 rpm for 30 min at 4°C to form a precipitate. The precipitate was dissolved in an appropriate amount of PBS solution and filtered five times through a 0.22 μm filter to obtain the EVs.

#### 2.2.2 Transmission electron microscopy morphology

Imaging was conducted using a JEM-1200EX transmission electron microscope at 100 kV. The procedure involved placing a sealing film on a glass slide and securing a copper grid with tweezers. A 10 μL aliquot of the sample was then applied to the copper mesh, ensuring the volume did not exceed the desired amount, and the excess liquid was gently removed with filter paper. Subsequently, a 10 μL drop of a 3% hydrogen peroxide acetate solution was added to the sample and incubated for 1–3 min. The excess stain was carefully blotted with filter paper. Finally, the grid was allowed to air dry for 10 min before proceeding with analysis. The imaging software used (4.6.0) is Olympus Image Shareware.

#### 2.2.3 Determination of particle size and potential

From the prepared sample of EVs, 100 μL of sample solution was added to the PBS solution, which was diluted fifty-fold to become the solution to be measured. Particle size and zeta potential of extracellular vesicles were analyzed using a Nano ZS90 Particle Size and Zeta Potential Analyzer equipped with a Nano Zetasizer ZS dynamic light scat-terometer. Measurements were conducted at 25°C. The average values were calculated by taking the mean of three measurements for each sample, with each measurement consisting of 100 runs.

### 2.3 Determination of the chemical composition of EVs

#### 2.3.1 Determination of total sugar content

1 mL of the EVs sample was polymerized at 10,000 rpm and the supernatant was discarded, after which 20 mL of anhydrous ethanol was added and sonicated at 60% power for 30 min. The mixture was centrifuged at 4,000 r/min for 10 min at room temperature to remove the solvent fraction. The remaining organic solvent was evaporated under nitrogen protection and the weight of the dry residue was measured, which represents the total sugar content.

#### 2.3.2 Determination of total lipid content

Centrifuge the collected EVs at 1000rpm for 30 min to facilitate sedimentation. The total lipid content of EVs was determined using the Bligh and Dyer method with methanol/chloroform extraction. Extract the 1 mL sedimented EVs sample with a mixture of methanol/chloroform (2:1, v/v; 3 mL). Add chloroform (1 mL) and ddH2O (1 mL) to the mixture, then vortex. Centrifuge the mixture at 2000g for 10 min at 22°C to obtain two phases (aqueous and organic). Collect the upper organic phase and evaporate the solvent at 60°C until dryness. Weigh the dried sample and measure the total lipid weight.

#### 2.3.3 Determination of protein content

Add 1 mL of EVs sample into 500 μL of RIPA buffer containing protease inhibitor (RIPA: FMSP = 100:1). Mix the EVs suspension with strong RIPA lysis buffer and vortex. Place EVs on ice for 30 min, during which repeatedly pipette the sample up and down using a pipette gun to ensure complete cell lysis. Centrifuge at 12,000 g for 5 min to collect the supernatant as the total protein solution. Determine the total protein content in the supernatant using the BCA protein assay. Reference the quantification method for exosomes and use EVs protein as a standard for subsequent experiments.

#### 2.3.4 Determination of total phenol and total flavonoid content

Triton X-100 at a concentration of 1 µL was added to 1 mL of EVs, followed by precipitation at 10,000 rpm and solubilization with 80% methanol to determine the total phenol and flavonoid contents. The total phenol content of EVs was assessed using the FolinCiocalteu method. Specifically, 0.3 mL of EVs was mixed with 4 mL of distilled water and 400 µL of Folin-phenol reagent. After incubating in the dark for 5 min, 2 mL of 5% Na_2_CO_3_ solution was added. The mixture was then allowed to react in the dark for 60 min, followed by measurement of the total phenol content using a spectrophotometer at 765 nm. For total flavonoid content, 0.5 mL of the EVs sample was mixed with 0.7 mL of distilled water and 0.2 mL of 5% NaNO_2_. After shaking well, the mixture was incubated in the dark for 6 min, followed by the addition of 0.2 mL of 10% Al(NO_3_)_3_. The mixture was shaken and left in the dark for another 6 min. Subsequently, 2 mL of 1 mol/L NaOH was added and mixed thoroughly. The final volume was brought to 5 mL with 1.4 mL of distilled water. After thorough mixing, the mixture was incubated in the dark for 6 min, and the absorbance was measured at 500 nm.

### 2.4 Measurement of chemical antioxidant capacity

To evaluate the chemical antioxidant activity of EVs, we employed three methods: ABTS, DPPH, and FRAP, using carbon trioxide as a reference standard. The results were expressed as carbon trioxide equivalents (TE) per milliliter (μmol/mL TE). For the ABTS antioxidant assay, a reaction mixture was prepared by combining 140 mmol/L K_2_S_2_O_8_ and 7 mol/L ABTS solution, followed by incubation for 12 h protected from light. The absorbance of the ABTS solution at 743 nm was adjusted to 0.70 ± 0.02 with anhydrous ethanol to obtain the final solution for determining the antioxidant properties of the samples. In the DPPH assay, DPPH powder was dissolved in 80% methanol to a concentration of 75 μmol/L. Each sample or Trolox solution (100 μL) was added to 3.80 mL of DPPH solution (75 μmol/L). The mixture was kept in the dark for 30 min, and the absorbance was measured at 517 nm. For the FRAP assay, a TPTZ solution was prepared by mixing 0.3 mol/L acetate buffer (pH 3.6), 20 mmol/L FeCl_3_ solution, and 10 mmol/L TPTZ work-ing solution in a 10:1:1 ratio. Then, 100 μL of the sample or Trolox solution was incubated with 3.90 mL of TPTZ solution for 30 min, protected from light, and the absorbance at 593 nm was measured.

### 2.5 Assessment of anti-inflammatory and antioxidant capacity

#### 2.5.1 Cell culture

The complete medium was prepared by combining 90% DMEM, 10% serum, and 1% penicillin/streptomycin. Frozen RAW264.7 macrophages were rapidly thawed in a 37°C water bath. Once the cell suspension reached 37°C, RAW264.7 macrophages were carefully aspirated into a 15 mL sterile centrifuge tube containing 9 mL of complete medium. The suspension was centrifuged at 1,000 rpm for 3 min at room temperature. The supernatant was discarded, and the RAW264.7 macrophages were resuspended in 1 mL of complete medium. The cell suspension was then gently transferred into culture flasks and shaken gently to ensure even distribution of the cells. After microscopic observation, the RAW264.7 macrophages were incubated in an incubator at 37°C with 80% relative humidity and 5% carbon dioxide.

#### 2.5.2 Cell viability assay

In 96-well cell culture plates, 10^4^ cells were seeded per well in 100 μL of complete medium and incubated for 24 h. Subsequently, the prepared EV stock solution was diluted with complete medium and applied to the RAW264.7 cell cultures at various concentrations. After 24 h of treatment, the wells were rinsed twice with 100 μL PBS. Finally, 10 μL of CCK-8 solution was added to each well, and the cells were incubated at 37°C for 2 h. Following incubation, the optical density (OD) was measured at 450 nm using a Thermo Scientific reader.

#### 2.5.3 Establishment of cellular stress models

Oxidative Stress Cell Model: A 0.03% solution of H_2_O_2_ was prepared by diluting a 3% H_2_O_2_ solution in complete medium. RW264.7 macrophages were seeded in 6-well plates, and 3 mL of the 0.03% H_2_O_2_ solution was added to each well. The cells were then incubat-ed at 37°C for 2 h to induce oxidative stress. LPS-Induced Cellular Inflammation Model: A solution of 2 mg/mL LPS was prepared by dissolving 2 mg of LPS in 1 mL of PBS. This solution was further diluted with complete culture medium to achieve a concentration of 3 μg/mL. Subsequently, the LPS-containing medium was added to a 6-well plate with cells, and the cells were treated and incubated for 4 h to establish the cellular inflammation model.

#### 2.5.4 Nitrogen monoxide (NO) determination

The NO assay kit and Griess A and B solutions were equilibrated to room temperature before use. Fifty microliters of the cell culture supernatant were transferred to each well of a 96-well plate. Subsequently, 50 μL of Griess A and B reagents were added sequentially to each well, with gentle mixing after each addition. The reaction was allowed to proceed, followed by measurement of absorbance at a wavelength of 540 nm.

#### 2.5.5 Malondialdehyde (MDA) determination

Using a cell spatula, transfer the cells from the 6-well plate into a 15 mL centrifuge tube and add 1 mL of extraction buffer per one million cells. After cell lysis, 100 μL of Sample Solution was combined with 300 μL of Reagent one following the instructions provided in the MDA kit. Centrifuge the mixture in a 95°C water bath for 30 min. Carefully transfer 200 μL of the supernatant to a new 96-well plate for absorbance analysis, with absorbance values measured at 532 nm and 600 nm.

#### 2.5.6 Glutathione (GSH) determination

Transfer the cells from the 6-well plate into a 15 mL centrifuge tube using a cell scraper, adding 1 mL of extraction buffer per 5 million cells. The cells were subjected to cell disruption in an ice-water bath using ultrasound at a power of 200 W, with 3-s pulses at 10-s intervals repeated 30 times. After centrifugation at 8,000 g for 10 min, the supernatant was collected for determining the GSH content. Following the instructions provided with the GSH assay kit, 20 μL of sample solution was mixed with 140 μL of Reagent 2 and 40 μL of Reagent 3. After thorough mixing, the mixture was incubated at room temperature for 2 min. Carefully transfer 200 μL of the supernatant to a new 96-well plate for absorbance analysis, measuring absorbance at 412 nm.

#### 2.5.7 Antioxidant enzyme index determination

The cells from the 6-well plate were collected into a 15 mL centrifuge tube, and 1 mL of cell extraction buffer was added for every 5 million cells. The mixture was thoroughly mixed to ensure complete extraction. Subsequently, the cytosolic fraction was kept on ice while subjected to disruption using ultrasound at 200 W power, with 3-s pulses at 10-s intervals repeated 30 times to disrupt the cells. After disruption, the sample was centrifuged at 8,000 g for 10 min at 4°C to obtain the supernatant. For the CAT and SOD enzyme activity assays, absorbance values were measured at 240 nm and 560 nm, respectively. For the GR enzyme activity assay, cells were suspended in 1 mL of assay cell extraction solution for every 5 million cells. Ultrasound treatment at 300 W power was applied with 3-s pulses at 7-s intervals repeated 30 times to facilitate cell disruption and intracellular component release. The sample was then centrifuged at 10,000 rpm for 10 min at 4°C to collect the supernatant. The GR enzyme activity was meas-ured using a 96-well UV plate to determine the OD value at 340 nm.

#### 2.5.8 Measuring the expression of inflammatory factors

300,000 cells were cultured per well in a 6-well plate. Cells were collected from the 6-well plate using a cell scraper and transferred into a 15 mL centrifuge tube. Centrifugation was carried out at 1,000 rpm for 3 min to pellet the suspended cells. The pelleted cells were washed twice with PBS and then treated with 500 µL of cell lysis buffer (TIANGEN, Beijing, China) for RNA extraction. The cDNA synthesis using the HiScript III All-in-one RT supermix reverse transcription master kit (Vazyme, Nanjing, China). Quantification was performed using the SYBR Green Pro Taq HS Premix qPCR kit (Vazyme, Nanjing, China). Primers specific for inflammatory genes were synthesized by UW Genetics (Shenzhen, China).

### 2.6 EVs loaded with nobiletin

#### 2.6.1 Preparation of EVs-nob

To prepare PLGA-nobiletin nanoparticles (PLGA-nob), 0.1 g of PLGA and 0.1 g of nobiletin were dissolved in dichloromethane by vortexing. The solution was then added dropwise to a 1% PVA solution while continuously stirring with a magnetic stirrer on ice. The mixture underwent sonication using a 200 W probe sonicator, alternating between 2 s of sonication and 2 s of pause, totaling 2 min. After centrifugation at 4°C and 8,000 rpm for 10 min, the supernatant was discarded. This centrifugation step was repeated twice, with the second round at 14,000 rpm for 40 min at 4°C. The resulting precipitate was redissolved in 0.5 mL of PBS to obtain PLGA-nobiletin nanoparticles. Subsequently, the prepared EVs were combined with PLGA-nobiletin nanoparticles at a 1:1 volume ratio and sonicated at 200 W for 5 min. The mixture was then centrifuged at 14,000 rpm for 40 min at 4°C. The supernatant was discarded, and the remaining pellet was redissolved in PBS to yield EVs-nob.

#### 2.6.2 Drug encapsulation and loading efficiency evaluation

The drug loading efficiency of EVs and the encapsulation rate of Nobiletin by PLGA was determined by Beckman Coulter UV- spectrophotometer. After being freezedried, the sample was weighed (10 mg) and then dissolved in a solution of ethanol:dichloromethane (10:1, v:v) at a concentration of 5 mL. The resulting mixture was sonicated for 10 min at 60% power, and subsequent to centrifugation at 8,000 *g* for 10 min, the supernatant was collected for further determination. The UV-spectrophotometer was employed to determine the maximum absorption wavelength of Nobiletin. The absorbance values at this wavelength were measured for different Nobiletin concentrations, and a standard curve was subsequently established. The encapsulation rate (ER) and loading capacity (LC) are calculated using the following equations:

$$\text{Encapsulation rate }\left(\text{ER}\right)=\left(1\;‐\text{ free content}\right)/\left(\text{input amount}\right)$$ (1)

$$\text{Loading capacity }\left(\text{LC}\right)=\left(\text{Extracellular vesicle drug content}\right)/\left(\text{Total mass of extracellular vesicles}\right)$$ (2)

### 2.7 Statistical analysis

The data were expressed as mean ± standard deviation from at least three replicates per sample. Statistical analyses including one-way analysis of variance (ANOVA) followed by Duncan’s test were conducted using SPSS 18.0 software, with a significance level set at *p* < 0.05 to assess statistical significance.

## 3 Results

### 3.1 Isolation and characterization of EVs from CRP

**FIGURE 1:**
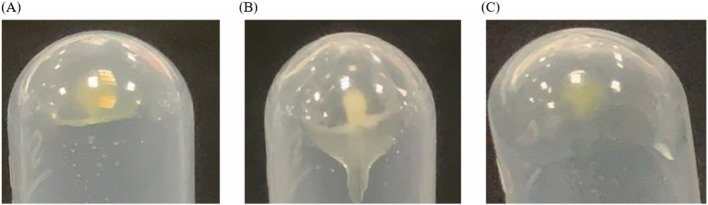
Extraction of CRP-derived extracellular vesicles. **(A)** EVs from 1-year-old CRP; **(B)** EVs from 2-year-old CRP; **(C)** EVs from 3-year-old CRP.

**FIGURE 2:**
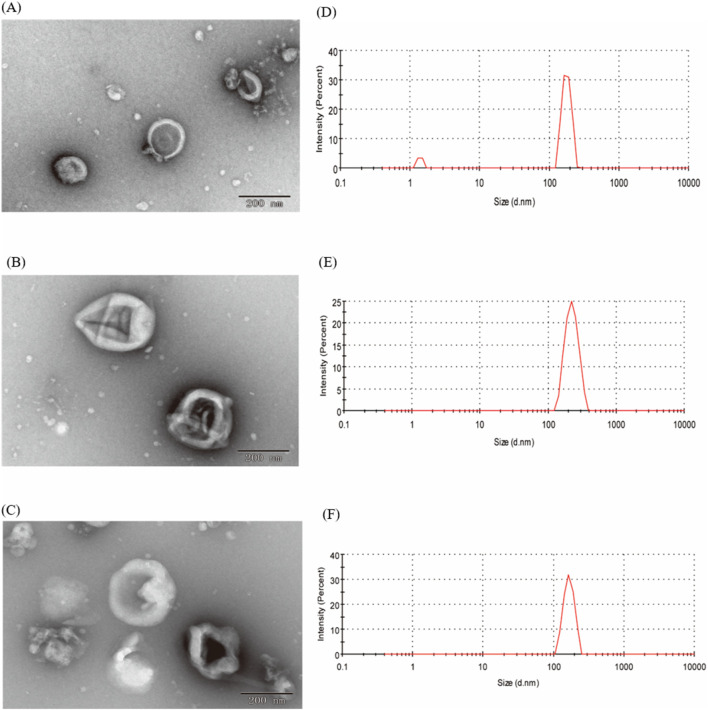
Shape and particle size of EVs. **(A)** TEM image of EVs from 1-year-old CRP; **(B)** TEM image of EVs from 2-year-old CRP; **(C)** TEM image of EVs from 3-year-old CRP; **(D)** Particle size distribution of EVs from 1-year-old CRP; **(E)** Particle size distribution of EVs from 2-year-old CRP; **(F)** Particle size distribution of EVs from 3-year-old CRP.

In our study, EVs from CRP stored under natural conditions for 1, 2 and 3 years were extracted and characterised by PEG polymer precipitation method (Figure 1). Transmission electron microscopy images showed that the size of EVs at different ageing stages was around 200 nm, and the central part of the vesicles was hollow, which was consistent with our previous findings (Li et al., 2023). The particle sizes of EVs aged for 1, 2 and 3 years CRP (named EVs1, EVs2 and EVs3) were 202 nm, 199.3 nm and 201.1 nm, respectively (Figures 2A–C), and the zeta potentials were measured to be −2.84 mV, −3.90 mV and −4.62 mV (Figures 2D, E). In addition, we observed that EVs3 had a more irregular shape compared to EVs1. irregular. This suggests that the spherical structure of the vesicles becomes increasingly unstable over time, leading to an increase in irregularity with age.

### 3.2 Chemical composition of EVs from CRP

**FIGURE 3:**
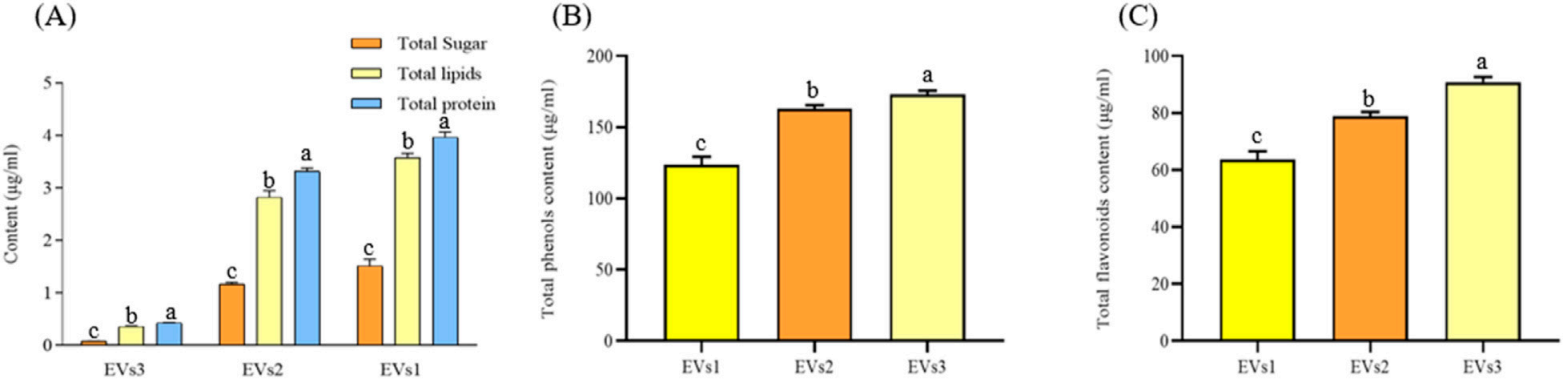
Chemical composition and content of different EVs. **(A)** Total sugar, lipid, and protein content of EVs1,EVs2 and EVs3; **(B)** Total phenolics content of EVs1,EVs2 and EVs3; **(C)** Total flavonoids content of EVs1,EVs2 and EVs3. Values in the same row with different superscript letters are significantly different (*p* < 0.05).

Sugars, lipids, and proteins are the main components of EVs. We quantified the total amounts of these components in the three different EVs (Figure 3). High levels of lipids (0.43–3.97 mg/mL) and proteins (0.35–3.57 mg/mL) were detected in all three types of EV samples, while the sugar content was comparatively low (0.084–1.51 mg/mL). The lipid and protein contents in EVs1 and EVs2 were similar and significantly higher than those in EVs3 (Figure 3A). This discrepancy may be attributed to the structural instability of EVs3, leading to the leakage of its contents.

The total phenol contents of EVs1, EVs2 and EVs3 were 123.49 ± 5.74 μg/mL, 162.93 ± 2.68 μg/mL and 172.94 ± 2.63 μg/mL, respectively (Figure 3B). The total flavonoid contents of EVs1, EVs2 and EVs3, although lower than the total phenol contents, were 63.52 ± 3.03 μg/mL and 78.93 ± 1.48 μg/mL and 90.48 μg/mL, respectively, The total flavonoid content of EVs1, EVs2 and EVs3, although lower than the total phenolic content, was 63.52 ± 3.03 μg/mL, 78.93 ± 1.48 μg/mL and 90.79 ± 1.82 μg/mL, respectively (Figure 3C). The significantly higher levels of total phenols and total flavonoids in EVs3 compared to EVs1 may be attributed to the increase in these compounds in CRP over time.

### 3.3 Antioxidant activity of EVs *in vitro*

The antioxidant activity of different EVs was evaluated using the ABTS, DPPH, and FRAP methods (Table 1). The antioxidant capacity of the EV samples was determined by their ability to scavenge ABTS+ and DPPH + radicals, and the absorbance values were recorded. The reducing capacity was assessed using the FRAP method, which measures the formation of a blue complex. The overall antioxidant capacity was then comprehensively evaluated using the APC score. The results indicated that the antioxidant capacity of EVs3 was stronger than that of EVs2 and EVs1. This may be attributed to the higher content of antioxidant substances in EVs3, enhancing its antioxidant capacity.

**TABLE 1:** Chemical antioxidant capacity of different EVs.

NO		Antioxidant activities (μmol/g TE)				
		ABTS	DPPH	FRAP	APC	Rank
1	EVs1	164.36 ± 0.77^c^	13.47 ± 0.09^c^	73.30 ± 0.05^c^	42.76	3
2	EVs2	241.24 ± 0.38^b^	32.50 ± 0.05^a^	143.42 ± 0.05^b^	81.26	2
3	EVs3	307.69 ± 0.38^a^	24.19 ± 0.12^b^	219.41 ± 0.10^a^	91.48	1

Note: ABTS, DPPH, and FRAP, are methods used to measure antioxidant activity, while APC, represents the total score of all three measurements combined. Rank shows the order of each sample’s overall antioxidant capacity. Values in the same row with different superscript letters are significantly different (*p* < 0.05).

### 3.4 Cytotoxicity evaluation of EVs

**FIGURE 4:**
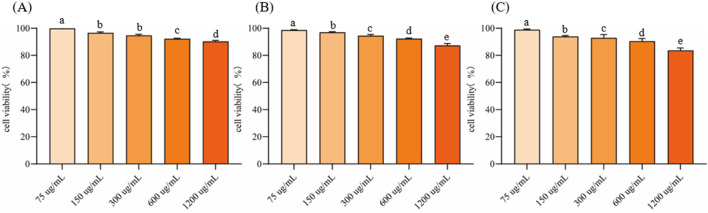
Effect of different EVs on RAW264.7 cell viability. **(A)** Cell activity in different concentrations of EVs1 treatment; **(B)** Cell activity in different concentrations of EVs2 treatment; **(C)** Cell activity in different concentrations of EVs3 treatment. Values in the same row with different superscript letters are significantly different (*p* < 0.05).

RAW264.7 cells were cultured to logarithmic growth phase, different concentrations of EVs were added and co-incubated in the cell culture incubator for 24 h. Cell viability was measured using the CCK-8 method to evaluate their cytotoxicity (Figure 4). The inhibition rate of all three EVs on RAW264.7 cells increased with the increase of the action concentration in a concentration-dependent manner. At a concentration of 75 μg/mL, the cell viability of EVs1, EVs2 and EVs3 treatments were 99.89% ± 0.66%, 98.66% ± 0.29% and 98.89% ± 0.52%, respectively (Figures 4A–C). When the concentration was increased to 300 μg/mL, the cell viability of EVs1, EVs2 and EVs3 treatments were higher than 90% with 94.73% ± 0.83%, 94.43% ± 0.93% and 92.84% ± 2.47%, respectively. When the concentration was increased to 1,200 μg/mL, the maximum cell viability was 90.28% ± 0.79% for EVs1 treatment, 87.32% ± 1.40% for EVs2 treatment and 83.65% ± 1.82% for EVs3 treatment. Since the cell viability of all three EVs treatments was higher than 90% at 300 μg/mL, this concentration was used for subsequent experiments.

### 3.5 Cellular antioxidant activity of EVs in RAW264.7 cell

#### 3.5.1 Effect of EVs on MDA、NO and GSH indicators

**FIGURE 5:**
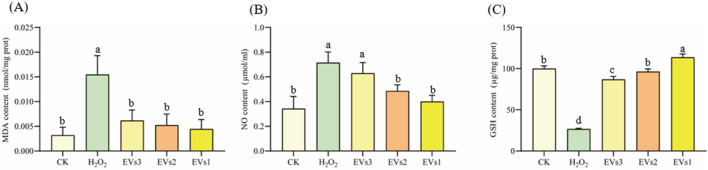
Impact of EVs treatment on intracellular antioxidant markers in RAW264.7 cells. **(A)** Malondialdehyde (MDA) content; **(B)** Nitric Oxide (NO) content; **(C)** Glutathione (GSH) content. Note: “prot” denotes protein. Values with differing superscript letters within the same row are significantly different (*p* < 0.05).

Oxygen radicals interact with the unsaturated fatty acids in lipids, leading to the production of oxidized lipids that break down into a complex series of compounds, including malondialdehyde (MDA). The MDA level is used as an indicator to assess the extent of oxidation occurring within the cell. The results show that MDA levels significantly increased in the H_2_O_2_ treated group compared to the blank control, indicating oxidative damage in the cells (Figure 5A). With the three EVs treatments, MDA levels in RAW264.7 cells were significantly reduced by 60.23%, 66.22%, and 71.34%, respectively, compared to the model group. In addition, there was no significant difference in MDA levels between the EVs-treated groups and the blank control group. These findings suggest that EVs treatment reduced membrane lipid peroxidation and prevented oxidative damage to cells.

NO detection kit was used to evaluate cell’s antioxidant capacity under different treatment conditions (Figure 5B). In the model group, NO content increased to 0.71 ± 0.09 μmol/mL after H_2_O_2_ treatment compared to the blank group. However, NO content decreased in the cells after treatment with the three EVs. While EVs3 treatment did not show a significant difference in NO content compared to the model group, both EVs2 and EVs1 treatments significantly reduced NO content, with reductions of 32.13% and 44.18%, respectively.

Glutathione (GSH) is a tripeptide composed of glutamine, cysteine, and glycine, and it is one of the most important antioxidants within cells. GSH plays a crucial role in protecting cells from oxidative stress and maintaining redox homeostasis. After being treated with H_2_O_2_, The level of GSH in cells was significantly decreased compared to the blank group (Figure 5C). However, after EV treatment, the intracellular GSH level gradually increased relative to the model group. Specifically, there was a 2.26-fold increase after EVs3 treatment, a 2.62-fold increase after EVs2 treatment, and a 3.28-fold increase after EVs1 treatment. These findings indicate that EV treatment effectively prevents the escalation of cellular oxidation levels and functions as an antioxidant. Notably, EVs1 demonstrates a particularly pronounced antioxidant effect.

#### 3.5.2 Effect of EVs on SOD、CAT and GR enzyme activity

**FIGURE 6:**
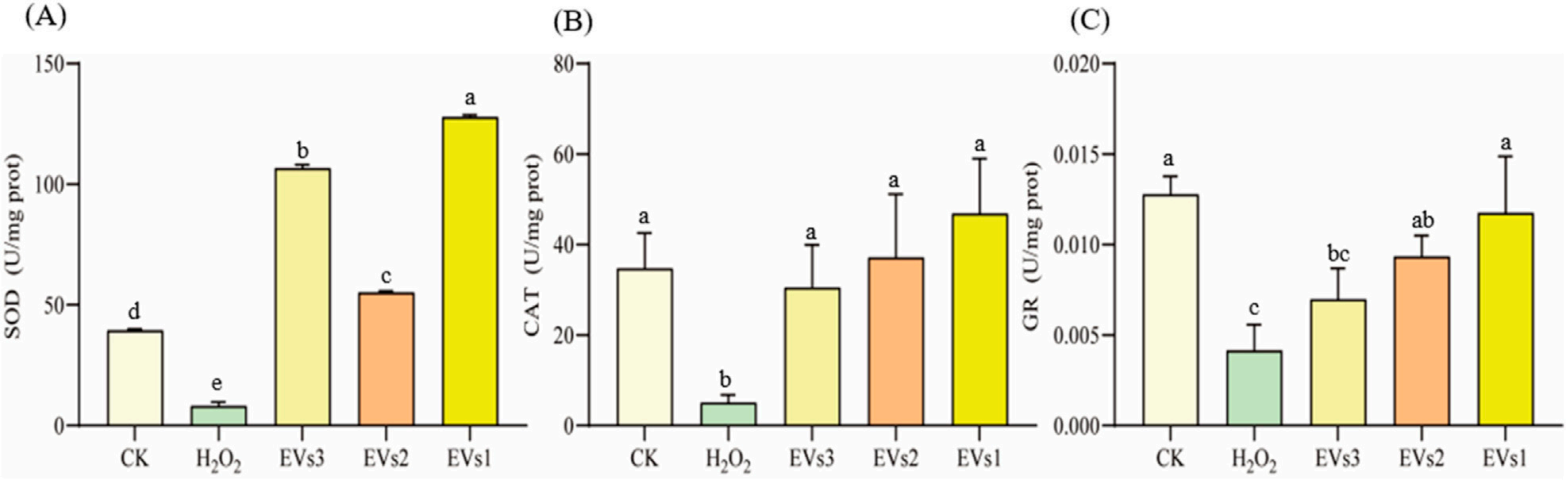
Impact of EVs treatment on intracellular antioxidant enzyme activity in RAW264.7 cells. **(A)** Superoxide dismutase (SOD) activity; **(B)** Catalase (CAT) activity; **(C)** Glutathione reductase (GR) activity. Note: “prot” denotes protein. Values with differing superscript letters within the same row are significantly different (*p* < 0.05).

The antioxidant enzyme system plays a pivotal role in maintaining redox homeostasis and counteracting free radicals primarily induced by oxidative stress. Superoxide dismutase (SOD), catalase (CAT), and glutathione reductase (GR) constitute key components of this system. We further assessed the impact of EV treatment on cellular SOD, CAT, and GR enzyme activities to gauge their antioxidant potential (Figure 6). Following H_2_O_2_ treatment, SOD activity significantly decreased compared to the blank group. However, co-incubation with the three EVs notably increased SOD activity. Specifically, it rose from 8.15 ± 1.68 U/mg in the model group to 106.65 ± 1.52 U/mg (EVs3), 55.22 ± 0.63 U/mg (EVs2), and 127.85 ± 0.79 U/mg (EVs1), indicating an enhancement in cellular SOD activity (Figure 6A). The effect of EVs treatment on CAT activity, demonstrating a significant increase. Compared to the model group, EV3, EVs2, and EVs1 treatments amplified CAT activity by 5.13-fold, 6.33-fold, and 8.23-fold, respectively (Figure 6B). Moreover, EV treatment significantly elevated GR activity (Figure 6C). At 300 μg/mL, EV treatment substantially increased GR activity compared to H_2_O_2_ treatment. GR activity rose from 0.0041 ± 0.0014 U/mg in the model group to 0.0070 ± 0.0017 U/mg (EVs3), 0.0093 ± 0.0012 U/mg (EVs2), and 0.010 ± 0.0031 U/mg (EVs1). These findings underscore the substantial impact of EVs on redox balance in RAW264.7 cells, elevating the antioxidant enzyme activity system and thereby enhancing cellular protection against oxidative damage.

### 3.6 Anti-inflammatory activity of EVs in RAW264.7 cell

#### 3.6.1 Effect of EVs on NO indicators after LPS treatment

**FIGURE 7:**
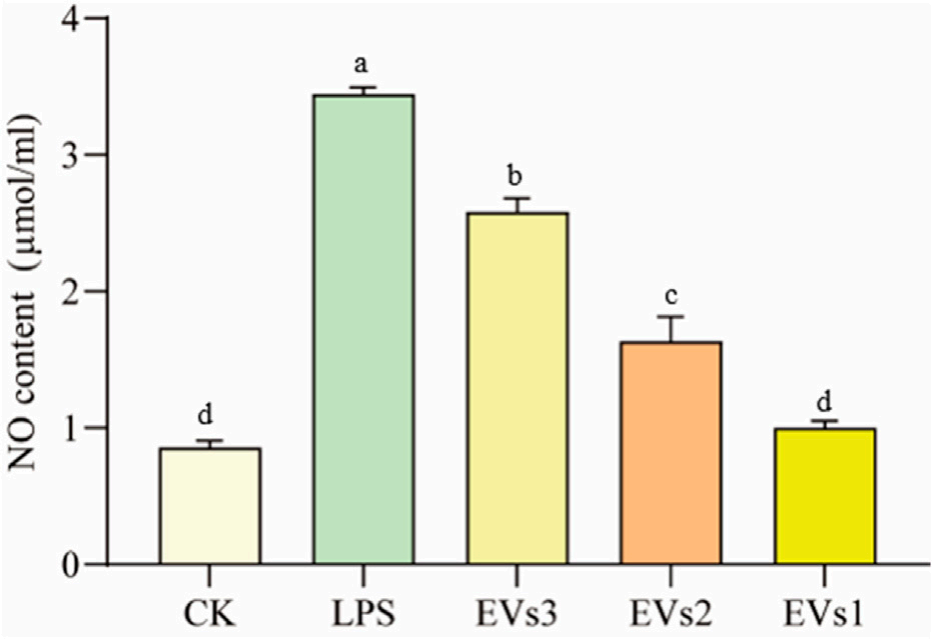
Impact of EVs treatment on NO content of RAW264.7 cells. Values in the same row with different superscript letters are significantly different (*p* < 0.05).

A cellular inflammation model was induced by treating RAW264.7 cells with LPS to evaluate the anti-inflammatory efficacy of EVs. The anti-inflammatory potential of the different EVs was assessed by measuring the NO content. The significant elevation in intracellular NO content following LPS treatment confirmed the successful establishment of the cellular inflammation model. All three EV treatments markedly decreased cellular NO content compared to the model group (Figure 7). Treatment with 300 μg/ml EVs resulted in reductions of 25.02% (EVs3), 52.54% (EVs2), and 70.89% (EVs1) in NO content, respectively. Notably, EVs1 exhibited the most pronounced effect on NO content reduction, indicating its potential to effectively mitigate the elevated NO levels induced by cellular inflammation and potentially offering protective effects against inflammation.

#### 3.6.2 Measurement of gene expression of inflammatory factors

**FIGURE 8:**
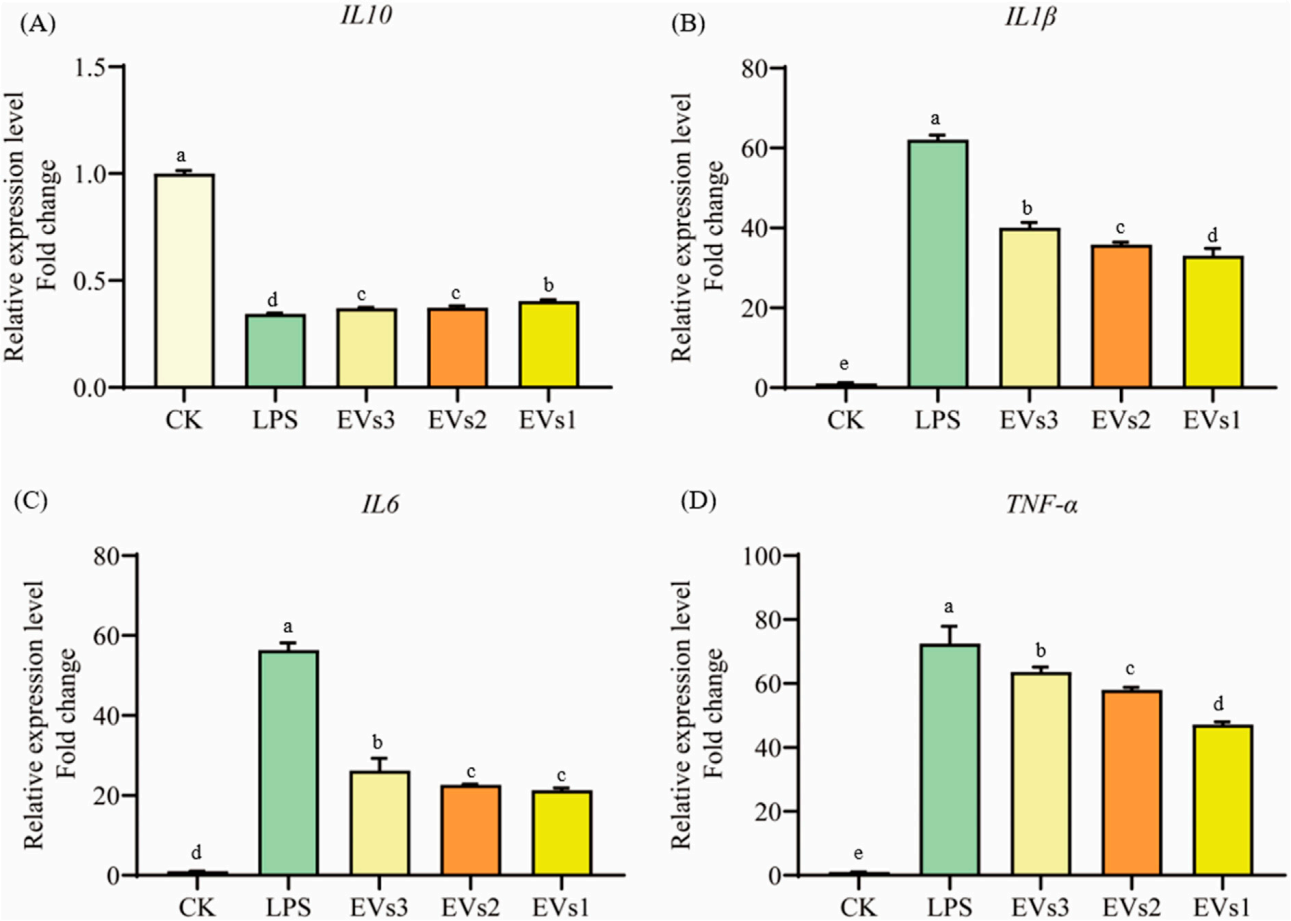
Impact of EVs treatment on gene expression level of RAW264.7 cells. **(A)** *IL-10* gene expression level; **(B)** *IL-1β* gene expression level; **(C)** *IL-6* gene expression level; **(D)** *TNF-α* gene expression level. Values in the same row with different superscript letters are significantly different (*p* < 0.05).

Cytokines are signaling molecules that mediate and regulate immunity, inflammation, and hematopoiesis. Changes in cellular inflammation levels can be assessed by measuring the expression of cytokine-related genes. In this experiment, LPS was used to induce inflammation in RAW264.7 cells to evaluate the anti-inflammatory effects of EVs at the cellular level (Figure 8). The results indicate that LPS treatment caused a significant decrease in the expression of the anti-inflammatory cytokine *IL-10* and a significant increase in the expression of the pro-inflammatory cytokines *IL-6*, *IL-1β*, and *TNF-α* compared to the blank group (Figure 8). However, treatment with EVs reversed these changes. Specifically, compared to the model group, EVs1 treatment increased the expression of *IL-10* by 0.17-fold and reduced the expression of *IL-6*, *IL-1β*, and *TNF-α* by 46.73%, 62.20%, and 34.90%, respectively (Figure 8). These findings suggest that EVs have potential therapeutic effects on cellular inflammation by modulating the expression of key cytokines.

### 3.7 Preparation and biological activity evaluation of EVs-nob nanoparticle

#### 3.7.1 Characterization of EVs-nob nanoparticle

**FIGURE 9:**
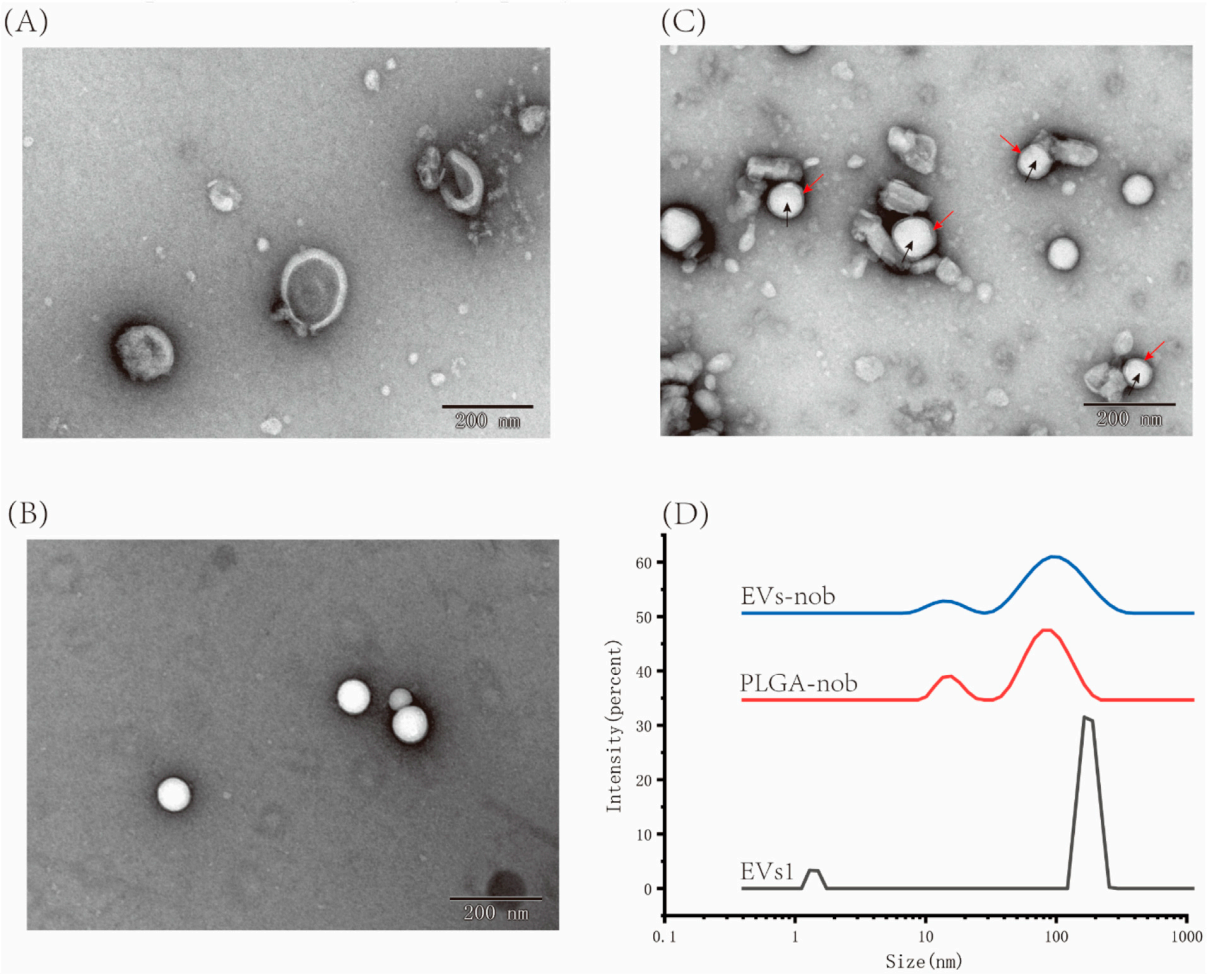
Characterization of EVs-nob nanoparticle. **(A)** Nano-scale morphology of EVs1; **(B)** Nano-scale morphology of PLGA-nob; **(C)** Nano-scale morphology of EVs-nob; **(D)** Particle size analysis of different nanoparticles. The black arrows indicate the PLGA-nob nanoparticles and the red arrows indicate the membrane structure of the outer layer of PLGA-nob.

By evaluating three types of EVs, we determined that EVs1 has an intact and stable morphology, as well as excellent antioxidant and anti-inflammatory properties (Figure 9A). Therefore, we used an ultrasound-assisted method to construct EVs-nob by combining EVs1 with PLGA-nob nanoparticles in a 1:1 ratio (v:v) (Figure 9). TEM results showed that the PLGA-nob nanoparticles were in a regular spherical shape with a smooth surface (Figure 9B). The successfully prepared EVs-nob nanoparticles were confirmed by comparison of TEM images through a continuous extrusion procedure to obtain the PLGA-nob nanoparticles encapsulated by EVs film. The formation of EVs-nob was demonstrated by the TEM image of EVs-nob, which showed that the white regular particles in the image were PLGA-nob with a grey-rendered wrapping layer on its surface. (Figure 9C). Particle size analysis of the nanoparticles showed that the PLGA-nob had a particle size of 92.93 nm, whereas the EVs-nob had a particle size of approximately 110 nm, a PDI coefficient of 0.436 and a zeta potential of −4.98 mV (Figure 9D). This may be due to the fact that the EVs were wrapped around the surface of the PLGA-nob through a constant extrusion process, which led to an increase in their particle size.

The nobiletin monomer samples were diluted to a concentration range of 2.5–40 μg/mL, and the absorbance values were measured using UV spectrophotometry. The linear relationship between nobiletin concentration and absorbance was determined to be y = 0.0524x + 0.0577. This linear regression equation can be used to calculate the solubility of nobiletin at any given concentration based on absorbance values. The amount of precipitated nobiletin and PLGA after loading was measured using UV-Vis spectrophotometry. Similarly, the amount of free nobiletin after centrifugation was calculated using the same method. According to the formula provided in Section 2.6.2, the encapsulation efficiency of nobiletin by EVs was determined to be 83.75% ± 2.83% (Equation 1). Based on this formula, the total mass of EVs (expressed as protein content) relative to the amount of loaded nobiletin represents the drug loading capacity of EVs-nob, which was calculated to be 2.79% ± 0.02% (Equation 2).

#### 3.7.2 Antioxidant evaluation of EVs-nob nanoparticle

**FIGURE 10:**
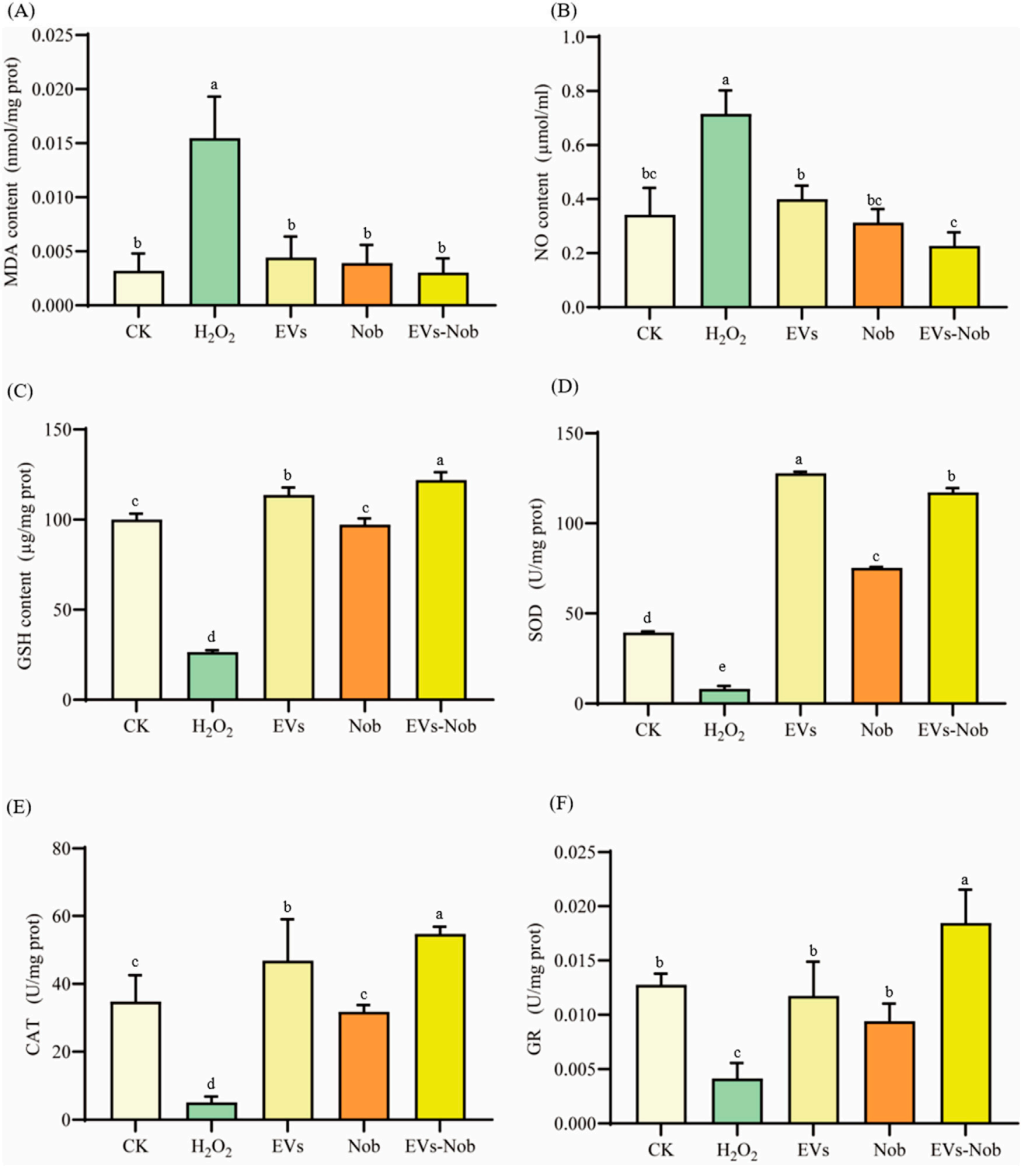
Impact of EVs-nob treatment on antioxidant indicators of RAW264.7 cells. **(A)** MDA content; **(B)** NO content; **(C)** GSH content; **(D)** SOD activity; **(E)** CAT activity; **(F)** GR activity. Values in the same row with different superscript letters are significantly different (*p* < 0.05).

This study evaluated the effect of EV membrane wrapping on the antioxidant capacity of Nobiletin by comparing 300 μg/mL concentrations of EVs, EVs-nob, and Nob at the same dose as EVs-nob (Figure 10). The antioxidant capacity was assessed by measuring the levels of MDA, NO, and GSH. The results indicated that the EVs-nob group significantly decreased MDA and NO levels while increasing GSH levels compared to both the EVs and Nob groups (Figures 10A–C). Specifically, EVs-nob treatment reduced MDA and NO levels by 22.10% and 27.52%, respectively, and increased GSH levels by 0.25-fold compared to the Nob group. Additionally, EVs-nob treatment enhanced antioxidant enzyme activity, with increases of 0.56-fold, 0.72-fold, and 0.96-fold in SOD, CAT, and GR activities, respectively, compared to the Nob group (Figures 10D–F). These findings suggest that coating Nob with EV membranes significantly enhances its antioxidant activity, making EVs-nob more potent than Nob or EVs alone.

#### 3.7.3 Anti-inflammatory evaluation of EVs-nob nanoparticle

**FIGURE 11:**
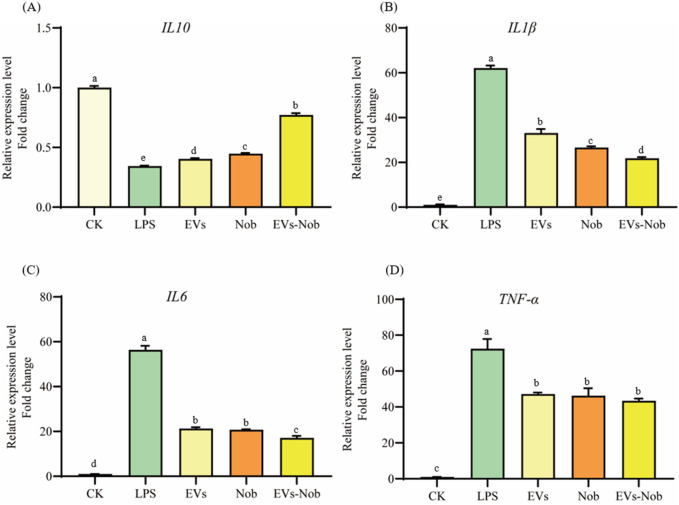
Impact of EVs-nob treatment on gene expression of RAW264.7 cells. **(A)** *IL-10* gene expression; **(B)** *IL-1β* gene expression; **(C)** *IL-6* gene expression; **(D)** *TNF-α* gene expression. Values in the same row with different superscript letters are significantly different (*p* < 0.05).

In the LPS-induced cellular inflammation model, the gene expression levels of *IL-6*, *TNF-α*, and *IL1-β* were notably lower in the EVs-nob group compared to both the Nob and EVs-treated groups (Figure 11). Relative to the Nob group, the EVs-nob group exhibited reductions of 17.75%, 6.27%, and 18.02% in *IL-6*, *TNF-α*, and *IL1-β* gene expression, respectively (Figures 11B–D). Conversely, the gene expression of the anti-inflammatory factor *IL-10* was significantly elevated (Figure 11A). These findings suggest that EVs-nob may offer enhanced efficacy in treating LPS-induced cellular inflammation.

## 4 Discussion

Extracellular vesicles (EVs) are small, membrane-bound particles released by cells that facilitate intercellular communication by transporting a variety of bioactive molecules, such as proteins, lipids, and nucleic acid (Colombo et al., 2014; van Niel et al., 2018). These vesicles play pivotal roles in regulating physiological and pathological processes, contributing to immune modulation, inflammation reduction, and tissue repair, thus promoting overall human health (Fusco et al., 2024). Notably, plant-derived EVs have shown significant health benefits. For instance, EVs extracted from ginger are efficiently taken up by intestinal macrophages and stem cells, where they activate nuclear factor E2-related factor 2 (Nrf2) and stimulate anti-inflammatory pathways, thereby supporting intestinal homeostasis (Liu et al., 2022). Grape-derived EVs have demonstrated the ability to target intestinal stem cells, promoting epithelial cell regeneration and aiding in the prevention and treatment of colitis (Ju et al., 2013). Broccoli-derived EVs exhibit anti-inflammatory properties by suppressing the expression of pro-inflammatory cytokines such as *TNF-α*, *IL-17A*, and *IFN-γ*, while enhancing the expression of the anti-inflammatory cytokine *IL-10*, offering therapeutic potential in inflammatory diseases (You et al., 2021). These natural, bioactive vesicles offer innovative solutions for disease prevention and treatment, making them valuable assets in the development of novel therapeutic strategies.

Chinese herbal medicine has a storied tradition in China and East Asia, with its origins traceable to ancient times. The rich repository of bioactive compounds found in Chinese herbs makes them excellent candidates for the extraction of plant extracellular vesicles. In this study, we utilized the PEG precipitation method to extract three types of EVs from CRP stored for 1 year (EVs1), 2 years (EVs2), and 3 years (EVs3). Transmission electron microscopy analysis indicated that these EVs were predominantly oval in shape with a hollow interior. This morphological characteristic is consistent with EVs derived from citrus fruits such as lemons and sweet oranges, as well as from medicinal herbs like ginseng and yam (Bruno et al., 2021; Wu et al., 2022; Xu et al., 2021; Yang et al., 2020). Notably, EVs3 exhibited a more irregular shape and contained more impurities, likely due to the extended storage period of the source material, which may result in tissue degradation and increased impurities. These findings imply that while EVs from various plant sources exhibit similar morphological features, storage duration can influence EV shape and purity. Quantitative analysis showed that all three types of EVs contained substantial amounts of lipids (0.43–3.97 mg/mL) and proteins (0.35–3.57 mg/mL), but relatively low levels of sugars (0.084–1.51 mg/mL). The low sugar content is attributable to the bilayered structure of EVs, composed mainly of membrane lipids and associated proteins, with sugars forming a minor component (Dad et al., 2021; Hao et al., 2024; Lian et al., 2022). Further analysis of bioactive substances revealed that all EVs were rich in total phenols and flavonoids. Given that EVs facilitate intercellular communication and material transport, their composition typically mirrors that of their source material (Chen et al., 2023; Colombo et al., 2014; Rome, 2019; Woith et al., 2021). The antioxidant capacity of the EVs was assessed using ABTS, DPPH, and FRAP assays. The results demonstrated that EVs3 possessed the highest antioxidant capacity, followed by EVs2, with EVs1 exhibiting the lowest capacity. This enhanced antioxidant activity in EVs3 can be attributed to its higher content of total phenols and flavonoids, which are well-known for their strong free radical scavenging properties (Bian et al., 2022; Cao et al., 2023). These findings underscore the potential impact of storage duration on the bioactive constituents and functional properties of plant-derived extracellular vesicles.

Plant-derived extracellular vesicles have shown significant health-promoting effects, particularly in their antioxidant and anti-inflammatory properties. EVs from medicinal and edible plants, as well as fruits, have demonstrated substantial potential in these areas (Hao et al., 2024; Zhang et al., 2023). For example, EVs extracted from apples, ginger, strawberries, and wheat have exhibited promising antioxidant and anti-inflammatory activities (Arai et al., 2021; Perut et al., 2021; Şahin et al., 2019; Zhang et al., 2016). Our current study found that CRP-derived EVs showed low toxicity effects on RAW264.7 cells, consistent with findings from other plant-derived EVs which also demonstrated low or no toxicity. This evidence underscores the safety profile of plant-derived EVs. In comparative studies using cell models, the three types of CRP-derived EVs effectively mitigated H_2_O_2_-induced oxidative stress and LPS-induced inflammation. The antioxidant activity of these EVs is attributed to their ability to modulate cellular antioxidant enzyme systems, enhancing the activity of enzymes such as superoxide dismutase (SOD), catalase (CAT), and glutathione reductase (GR), while reducing the levels of oxidative stress markers like malondialdehyde (MDA) and nitric oxide (NO). Additionally, they alleviate excessive cellular inflammation by reducing the expression of pro-inflammatory cytokines such as *IL-6*, *IL-1β*, and *TNF-α*.

Moreover, EVs serve as efficient nanocarriers for the delivery of bioactive substanc-es (Fan et al., 2022; López De Las Hazas et al., 2023; Shao et al., 2023). For example, lemon-derived EVs have been used to deliver therapeutic agents to tumour sites to inhibit gastric cancer growth by activating the ROS signalling pathway to induce cancer cell death (Yang et al., 2020). Additionally, acerola-derived vesicles have been employed to encapsulate and protect miRNA, facilitating targeted delivery to specific cells, which highlights the versatility of EVs in precision medicine (Umezu et al., 2021). Similarly, ginseng-derived exosomes have been shown to deliver microRNAs that stimulate the neural differentiation of stem cells, emphasizing their potential in regenerative medi-cine (Xu et al., 2021). In this experiment, we found that EVs possess good anti-inflammatory and antioxidant properties, making them suitable as novel antioxidants. To further explore the potential of EVs as natural antioxidants, we investigated the possibility of encapsulating Nobiletin in EVs. Electron microscopy images showed that EVs-nob exhibited good field cleanliness and dispersibility. It was also observed that the particle size of encapsulated EVs-nob was larger than that of the original PLGA-nob, which is consistent with the findings of other research groups (Li et al., 2023; Zhu et al., 2023). Additionally, we found that loading Nobiletin onto EVs enhanced their anti-inflammatory and antioxidant effects. This finding aligns with previous studies where researchers used grapefruit-derived extracellular vesicles to deliver bioactive substances, including curcumin and folic acid (Wang et al., 2013).

Extracellular vesicles (EVs) are an emerging field of research with a promising future. Currently, although we understand that the storage time of CRP affects the changes in vesicle structure and chemical composition of EVs, in addition to the excellent cellular antioxidant and anti-inflammatory activities of CRP-derived EVs, as well as their potential as nanocarriers for encapsulation of biologically active substances, there are still some shortcomings due to the preliminary nature of this research. (1) More examples are needed to explore the effects of some processing behaviours (e.g., storage time, preservation temperature, other light processing methods) on the structure, composition, and activity of EVs after plant harvesting. Research in this area could ensure the quality and stability of EVs extracted from plant sources; (2) the full impact of behaviours such as storage time on the contents of plant EVs needs to be further explored using metabolomic and proteomic techniques; and (3) in addition, in-depth explorations of the ability of plant-derived EVs to act as targeted drug delivery systems and their intrinsic mechanisms of cellular interactions are needed.

## 5 Conclusion

In conclusion, extracellular vesicles as EVs1, EVs2 and EVs3 were extracted from CRP stored for 1, 2 and 3 years, respectively, and characterised for their basic features and biological activities. The available results demonstrate that storage time affects the microstructure and chemical content of EVs in citrus plants. Increased storage time disrupted the structural integrity of the vesicle structure of plant EVs and caused changes in the characterised active components. In addition, the composition of EVs showed higher contents of total proteins (0.35–3.57 mg/mL) and lipids (0.43–3.97 mg/mL) and lower contents of total carbohydrates (0.084–1.51 mg/mL). Total phenols and flavonoids were found in EVs, where the overall concentration of flavonoids was lower than that of phenols. In cellular studies, EVs1 showed the strongest ability to inhibit H_2_O_2_-induced oxidative stress-related enzyme activities, demonstrating a strong cellular antioxidant capacity. In addition, EVs1 inhibited LPS-produced inflammatory cytokines (*IL-6*, *TNF-α*, and *IL-1β*) and possessed anti-inflammatory properties. Nobiletin was successfully encapsulated in EVs1 with an encapsulation efficiency of 83.75% ± 2.83% and a drug loading capacity of 2.79% ± 0.02%. After encapsulation, EVs-nob showed enhanced antioxidant and anti-inflammatory activities. In conclusion, our study revealed the composition, chemical composition, biological activities and possible uses of CRP-derived EVs with different storage times.

## Data Availability

The original contributions presented in the study are included in the article/supplementary material, further inquiries can be directed to the corresponding author.
